# Human Leptospirosis: Seroreactivity and Genetic Susceptibility in the Population of São Miguel Island (Azores, Portugal)

**DOI:** 10.1371/journal.pone.0108534

**Published:** 2014-09-25

**Authors:** Lisa M. Esteves, Sara M. Bulhões, Claudia C. Branco, Francisco M. Mota, Clara Paiva, Rita Cabral, Maria Luisa Vieira, Luisa Mota-Vieira

**Affiliations:** 1 Molecular Genetics and Pathology Unit, Hospital of Divino Espírito Santo of Ponta Delgada, EPE, São Miguel Island, Azores, Portugal; 2 Infectious Diseases Department, Hospital of Divino Espirito Santo of Ponta Delgada, São Miguel Island, Azores, Portugal; 3 Internal Medicine Department, Hospital of Divino Espirito Santo of Ponta Delgada, EPE, São Miguel Island, Azores, Portugal; 4 Leptospirosis and Lyme Borreliosis Group, Unit of Medical Microbiology, Instituto de Higiene e Medicina Tropical, Centro de Recursos Microbiológicos, Universidade Nova de Lisboa, Lisboa, Portugal; 5 Azores Genetics Research Group, Instituto Gulbenkian de Ciência, Oeiras, Portugal; 6 Centre for Biodiversity, Functional and Integrative Genomics, Faculty of Sciences, University of Lisboa, Lisboa, Portugal; Federal University of Pelotas, Brazil

## Abstract

**Background:**

Leptospirosis is a worldwide zoonotic and recognized neglected infectious disease. It has been observed that only a proportion of individuals exposed to pathogenic species of *Leptospira* become infected and develop clinically evident disease. Moreover, little information is available in subsequent reinfections. In the present study, we determine if a first infection with leptospirosis protects against subsequent reinfection, and investigate which of the host genetic factors are involved in the susceptibility and resistance to leptospirosis.

**Methodology and Findings:**

We conducted, in 2011, a retrospective hospital-based case-control study in the São Miguel Island population (Azores archipelago). In order to determine the seropositivity against pathogenic *Leptospira* after the first episode of leptospirosis, we performed a serological evaluation in 97 unrelated participants diagnosed with leptospirosis between 1992 and 2011. The results revealed that 46.4% of the 97 participants have circulating anti-*Leptospira* antibodies, and from these participants 35.6% maintained the seroprevalence for the same serogroup. Moreover, three of them were reinfected with unrelated *Leptospira* serovars. The genetic study was carried out by adding a control group composed of 470 unrelated healthy blood donors, also from São Miguel Island. Twenty five SNPs among twelve innate immune genes – *IL1α*, *IL1β*, *IL6*, *IL10*, *IL12RB1*, *TLR2*, *TLR4*, *TLR9*, *CD14, CISH, LTA* and *TNF* – were genotyped, as well as *HLA* class I (–*A* and –*B*) genes. Association analysis indicates that genotypes -511GG (OR = 1.6, 95%CI 1.01-2.56, p = 0.04) in *IL1β*, +1196CG (OR = 2.0, 95%CI 1.26-3.27, p = 0.003) in *IL12RB1*, -292TA (OR = 1.8, 95% CI 1.06–2.1, p = 0.03) and +3415CG (OR = 1.8, 95% CI 1.08–3.08, p = 0.02), both in *CISH* confer susceptibility to pathogenic *Leptospira*.

**Conclusion:**

The present study suggests some degree of long-term protection against leptospires with an attenuation of symptoms in case of reinfection. Moreover, our data supports the genetic influence of *IL1β*, *IL12RB1* and *CISH* genes and the susceptibility to leptospirosis infection.

## Introduction

Leptospirosis is a worldwide zoonotic and recognized neglected infectious disease, caused by spirochetes of the *Leptospira* genus from the family Leptospiraceae [1]. This disease is known for its endemicity, and is considered a public health problem, due to its high annual incidence rate in semi-tropical climates, such as the Azores Islands (Portugal) [2]. This archipelago is composed of nine islands that constitute a unique environment to investigate the interactions between hosts and pathogens overtime. In São Miguel and Terceira Islands, the pathogenic leptospires are responsible for severe human disease leading to a systemic infection, characterized by clinical manifestations that vary greatly from flu-like symptoms to multiple organ failure and death. The disease progression is influenced, in part, by the production of circulating antibodies directed against serovar specific lipopolysaccharides (LPS), by the dose of infecting inoculum, and by the virulence characteristics of the infecting strain [3], [4].

The study of the genetic susceptibility to infectious disease has undergone revolutionary change over the last decade. However, not much is known about the host's genetic variation in the innate immune response to pathogenic *Leptospira* species. From our knowledge, there are only two previous studies that explore the genetic polymorphisms and the susceptibility to leptospirosis. The first study, conducted by Lingappa and collaborators, associated the human leukocyte-like antigen DQ-6 (HLA-DQ6) to an increased risk to leptospirosis among triathletes who ingested contaminated water [5]. In the second study, Fialho and colleagues reported that alleles from *HLA-A* (*24 and *31) and HLA-B*08, as well as alleles from the interleukin 4 (*IL-4*) and *IL-4Rα* genes were present in significantly higher frequencies in patients with a history of leptospirosis from Terceira Island in the Azores archipelago [6].

The major source of information concerning host immune response to leptospirosis was obtained from experimental animal models and human cells. Results indicate that, in mouse models, leptospiral lipoproteins and LPS stimulate Toll-like receptors (TLRs) – in particular TLR4 (the receptor for bacterial lipopolysaccharide) and TLR2 (which recognizes a wide variety of microbial ligands) [7]. On the other hand, in human cell models, leptospiral LPS activates TLR2 rather than the TLR4, indicating that the relative disease resistance of mice is linked to differential sensing of LPS by TLR4 [8], [9]. However, more recently, it was demonstrated that TLR2, as well as TLR4 and TLR5, play a role in the response to viable pathogenic leptospires in a human whole blood model [10]. These contradictory results open the possibility to investigate TLR polymorphisms and human leptospirosis susceptibility.

Another aspect to take in consideration is that infectious diseases are complex traits; therefore, other genes outside the innate immune genes may influence host susceptibility. Recently, Khor and colleagues (2010) observed strong associations between variant alleles in *CISH* (multiple cytokines inducible SH2-containing protein) gene and increased susceptibility to tuberculosis bacteremia and malaria parasitemia [11]. For this reason, it is of interest to evaluate the effect of these variants in leptospirosis susceptibility. In the present study, we investigated whether a first infection by leptospires protects against subsequent reinfection in high-risk human populations, in order to evaluate cross-protective immunity against leptospirosis, and determine the possible associations of variants in candidate genes of the innate immune system with susceptibility to human leptospirosis.

## Methods

### Ethics statement

The project follows the international ethical guidelines and was approved by the Ethics Committee for Health of the Hospital of Divino Espirito Santo of Ponta Delgada, EPE (HDES). The study design includes, from all participants, written informed consent, confidentiality and an abandonment option in case of expressed will. From those who freely accepted to participate, two blood samples were collected into EDTA (7.5 ml) and dry tubes (4.9 ml) for DNA extraction and serum separation, respectively. The control population consists of 470 DNA samples of unrelated healthy blood donors from São Miguel Island selected from the anonymized Azorean DNA bank, which was built according to the international ethical guidelines for sample collection, processing and storage [12].

### Study design and participants

The present work is a retrospective hospital-based case-control study. We invited 97 unrelated individuals − cases group − that attended the HDES, between 1992 and 2011, and were clinically diagnosed and/or MAT positive (agglutination titres ≥1∶160), and treated for leptospirosis to participate in the study. From all participants, we collected two blood samples and epidemiological information, such as demographic data signs and symptoms of leptospirosis. We were also able to retrieve, from the time each participant was hospitalized, the retrospective MAT (microscopic agglutination test) results [13]. The clinical history was completed by individual questionnaire, which was elaborated in the scope of the research project “Leptospirosis in Azores” (Epidemiology and Control of Leptospirosis in Azores Islands, São Miguel and Terceira 2003–2008) [14].

### Sample processing for serology and genotyping

Unique serum samples were aliquoted and stored at −80°C for further detection of anti-*Leptospira* antibodies, using MAT at the Leptospirosis laboratory of the Instituto de Higiene e Medicina Tropical (IHMT, Lisbon). MAT was performed using a battery of 25 live pathogenic serovars (including four local circulating strains) representative of 15 serogroups, and a saprophytic serovar of *L. biflexa* as an internal control. Samples were initially screened at 1∶40 dilution, and the reacting sera were further double-titrated to the end point, defined as the highest dilution that agglutinated 50% or more of the leptospires. The samples were considered positive when agglutination titres were equal or above 1∶160, undetermined when titres were bellow the cut-off 1∶160, and negative when no agglutination titres were observed.

Human genomic DNA from cases was extracted from whole blood, using the QIAamp Blood mini kit (Qiagen) protocol. The DNA was quantified by UV spectrometry and diluted to a 25 ng/µl working solution to perform genotyping by real time PCR and Snapshot multiplex PCR. To genotype *HLA–A* and *–B* genes, DNA was diluted to a 30 ng/µl working solution.

### SNP genotyping

Genetic variants were chosen considering their association to leptospirosis and to other infectious diseases, as well as their immune response to pathogens (Table S1). In total, 25 variants among 12 candidate genes were genotyped by three Snapshot Multiplex PCR panels, described by Esteves et al. [15] and singleplex real-time PCR techniques. The genotyping of *CISH* (rs6768330, rs622502, rs2239751 and rs414171), *TNF* (rs1800629 and rs361525), *IL10* (rs1800871) and *TLR9* (rs5743836) was performed in a 7500 fast real-time PCR system (Life technologies). Amplification of the *TLR2* insertion-deletion polymorphism was achieved under the conditions previously described [16], [17]: initial denaturation step at 95°C for 5 min, then 35 cycles at 94°C for 1 min, 58°C for 1 min and 72°C for 1 min, followed by final extension at 72°C for 10 min. The amplified products were separated on an ABI 3130 Genetic Analyzer (Life technologies), and fragment analysis was performed with GeneMapper software version 3.7 (Life technologies). The primers and probes (Table S2) were synthesized by two suppliers: Life technologies (rs6768330, rs622502, rs2239751, rs361525, rs1800871 and *TLR2* indel) and TibMolBiol (rs414171, rs6768330 and rs1800630). Since the sequence designed for rs1800630 was unsuccessful, we genotyped a statistically identical SNP (siSNP) rs2844482 in complete LD (r^2^ = 1.0) with rs1800630, as described by Zhao et al. (2007) [18]. The genotyping of *HLA* class I genes (*–A* and *–B*) was performed by PCR-SSP Olerup SSP (GenoVision Inc.), according to the manufacturer's instructions, in the 97 cases. PCR products were visualized after electrophoresis on a 4% agarose gel stained with SYBR Green, followed by HLA allele identification using the Helmberg-SCORE software version 3.320T (Olerup SSP AB, Saltsjöbaden, Sweden). As controls, we selected 106 individuals, from the 470 unrelated healthy control group, previously genotyped for *HLA* class I genes in São Miguel Island population [19].

### Statistic analysis

Allele frequencies were calculated by direct counting. Statistical analyses were performed with Arlequin software package, version 3.5, to calculate the Hardy-Weinberg equilibrium, gene diversity and haplotypes. Analysis of pairwise linkage disequilibrium between SNPs was carried out using the statistics D′ and correlation coefficient with Haploview software, version 3.2. The odds ratio (OR) and respective 95% confidence interval (CI) were calculated using a 2-way contingency table analysis [20]. The difference was considered to be statistically significant when p<0.05.

## Results

### Demographic, clinical and laboratory characterization of leptospirosis participants

Participant's characteristics are described in Table 1. Mean age of cases was 44.1 (±16.2) years. The presence of male participants (89.7%) exceeded that of females (10.3%), and 38.1% of the participants are farmers. At the time of infection, participants presented a set of common flu-like symptoms: myalgia (96.9%), fever (94.9%), chills (84.5%) and headache (76.3%). Only two cases of meningitis were reported. The most frequently observed alterations in laboratory parameters, in the 97 participants, were elevated hepatic enzymes – alanine transaminase (52.6%) and alkaline phosphatase (51.6%) –, bilirubin (32.4%), thrombocytopenia (49.5%) and leukocytosis (37.1%).

**Table 1:** Demographic, clinical and laboratory characterization of the 97 leptospirosis participants.

	Participants			Participants	
Demographic characterization	N = 97	(%)	Clinical data	N = 97	(%)
**Age at infection (yr)**			Myalgia	94	(96.9)
Mean	44.1±16.2	–	Fever	92	(94.9)
Range	16–86	–	Chills	82	(84.5)
**Sex**			Headache	74	(76.3)
Male	87	(89.7)	Elevated ALT/ASP^a^	51	(52.6)
Female	10	(10.3)	Elevated ALP/GGT^b^	50	(51.6)
**Profession**			Thrombocytopenia	48	(49.5)
Farmer	37	(38.1)	Jaundice	39	(40.2)
Retired	17	(17.5)	Leucocytosis/neutrophily	36	(37.1)
Housewife	6	(6.2)	Coluria/bilirrubin	32	(32.4)
Greenhouse workers	5	(5.2)	Nausea	31	(32.0)
Bricklayer	4	(4.1)	Urea/creatinine	28	(28.9)
Others	28	(28.9)	Vomiting	22	(22.7)
**Municipalities of São Miguel Island**			Elevated CK^c^	22	(22.7)
Ponta Delgada	54	(55.7)	Anorexy	22	(22.7)
Ribeira Grande	23	(23.7)	Prothrombin	15	(15.5)
Lagoa	9	(9.3)	Cough	13	(13.4)
Vila Franca do Campo	7	(7.2)	Dyspenia	13	(13.4)
Nordeste	3	(3.1)	Hipoxemia	13	(13.4)
Povoação	1	(1.0)	Anemia	11	(11.3)
			Odynophagy	9	(9.3)
			Diarrhea	5	(5.2)
			Hemoptysis	5	(5.2)
			Cilindruria	4	(4.1)
			Conjunctivitis	4	(4.1)
			Petechiae	3	(3.1)
			Meningitis	2	(2.1)

a ALT: Alanine transaminase/ASP: Aspartate transaminase.

b ALP: Alkaline phosphatase/GGT: Gamma glutamyltransferase.

c Kreatinine Kinase.

The presence of persistent anti-*Leptospira* antibodies was investigated by MAT on the serum samples collected in 2011. From the 97 participants, 45 (46.4%) contained significant levels of anti-*Leptospira* antibodies, 32 (33.0%) were negative, and 20 (20.6%) had a borderline result, *i.e.*, a specific reactivity but below the threshold of positivity at 1∶160 (titre assumed by the reference laboratory for endemic areas) (Table 2). To clarify whether a first infection may or may not contribute to protect against a subsequent reinfection, we compared the serological results obtained at the time of infection with those collected in 2011 within the 45 positive cases (Table 3). We observed that 16/45 (35.6%) individuals maintained anti-*Leptospira* antibodies from the same presumptive serogroup, although in 13/16 (81.3%) of them with decreased titres. Moreover, 11/45 (24.4%) individuals showed seroreactivity against leptospires of the serogroup with the highest titre, among others equally reactive during the first infection. Table 3 also shows the inverse situation, in 2011 six individuals (13.3%) evidenced a co-agglutination with leptospires from more than one serogroup, one of which overlapping to the initially registered. Three individuals – ID numbers 12, 97 and 103 – revealed distinct presumptive serogroups in both collection periods, suggesting that an asymptomatic leptospirosis reinfection occurred since the first episode. In addition, Table 3 also evidences that, in 2011, the most common serogroups belong to Icterohaemorrhagiae (73.3%) and Ballum (6.67%) being the remaining 20% attributed to co-agglutinations. From the HDES clinical records, we found that no affected children attended the Hospital with suspected leptospirosis; the youngest person was 16 years old (participant in the present study), which suggests that, perhaps, there are age-dependent changes in innate and adaptive immune response to *Leptospira* infection, as previously suggested [21], [22].

**Table 2:** MAT serological data obtained in 2011, after *n* years of first confirmed diagnosis.

		Participants						
			MAT (Antibodies against *L. Interrogans* s.l.)					
			Positive		Negative		Not Determined	
Year of leptospirosis diagnosis (Yr)	Years after first confirmed diagnosis (N)	Total (N)	N	(%)	N	(%)	N	(%)
1992	19	2	0	(0.0)	1	(50.0)	1	(50.0)
1993	18	1	0	(0.0)	1	(100)	0	(0.0)
1994	17	3	2	(66.6)	0	(0.0)	1	(33.3)
1995	16	4	1	(25.0)	0	(0.0)	3	(75.0)
1996	15	4	0	(0.0)	3	(75.0)	1	(25.0)
1997	14	3	3	(100)	0	(0.0)	0	(0.0)
1998	13	5	3	(60.0)	1	(20.0)	1	(20.0)
1999	12	1	0	(0.0)	1	(100)	0	(0.0)
2000	11	6	3	(50.0)	2	(33.3)	1	(100)
2001	10	4	2	(50.0)	1	(25.0)	1	(25.0)
2002	9	7	2	(28.6)	4	(57.1)	1	(14.3)
2003	8	10	5	(50.0)	2	(20.0)	3	(30.0)
2004	7	12	4	(36.4)	6	(54.5)	2	(9.10)
2005	6	11	4	(40.0)	3	(30.0)	4	(30.0)
2006	5	3	3	(100)	0	(0.0)	0	(0.0)
2007	4	5	1	(20.0)	4	(80.0)	0	(0.0)
2008	3	5	4	(80.0)	1	(20.0)	0	(0.0)
2009	2	2	2	(100)	0	(0.0)	0	(0.0)
2010	1	6	3	(50.0)	2	(33.3)	1	(16.7)
2011	0	3	3	(100)	0	(0.0)	0	(0.0)
**Total**	**–**	**97**	**45**	**(46.4)**	**32**	**(33.0)**	**20**	**(20.6)**

**Table 3:** Serological evaluation: comparison of Microscopic Agglutination Test (MAT) positive results (retrospective and in 2011).

Year of infection	Participant ID	MAT – Retrospective serological evaluation		MAT – Results in 2011 (unique samples)	
		Presumptive serogroup	MAT titre	Presumptive serogroup	MAT titre
1994	13	Icterohaemorrhagiae	1∶1280	Icterohaemorrhagiae	1∶320
1994	52	Icterohaemorrhagiae	1∶160	Co-agglutination (Icterohaemorrhagiae; Australis; Ballum)	≤1∶320
1995	17	Icterohaemorrhagiae	1∶10240	Co-agglutination (Icterohaemorrhagiae; Tarassovi)	1∶160
1997	43	Icterohaemorrhagiae	1∶640	Icterohaemorrhagiae	1∶160
1997	48	Icterohaemorrhagiae	1∶1280	Icterohaemorrhagiae	1∶320
1997	81	Co-agglutination (Icterohaemorrhagiae; Canicola)	≤1∶2560	Icterohaemorrhagiae	1∶320
1998	14	Icterohaemorrhagiae	1∶1280	Co-agglutination (Icterohaemorrhagiae; Ballum)	1∶160
1998	16	ND	NA	Icterohaemorrhagiae	1∶160
1998	20	ND	NA	Icterohaemorrhagiae	1∶1280
2000	97	Canicola	1∶320	Icterohaemorrhagiae	1∶320
2000	103	Co-agglutination (Australis; Pomona)	≤1∶640	Icterohaemorrhagiae	1∶160
2000	104	Co-agglutination (Icterohaemorrhagiae; Canicola)	1∶320	Icterohaemorrhagiae	1∶320
2001	11	Co-agglutination (Icterohaemorrhagiae; Ballum)	≤1∶5120	Icterohaemorrhagiae	1∶320
2001	32	Co-agglutination (Icterohaemorrhagiae; Tarassovi)	≤1∶1280	Icterohaemorrhagiae	1∶160
2002	63	Co-agglutination (Icterohaemorrhagiae; Canicola; Cynopteri)	1∶1280	Co-agglutination (Icterohaemorrhagiae; Cynopteri)	≤1∶640
2002	50	Icterohaemorrhagiae	1∶1280	Icterohaemorrhagiae	1∶1280
2003	55	Icterohaemorrhagiae	1∶1280	Icterohaemorrhagiae	1∶640
2003	67	Icterohaemorrhagiae	1∶640	Icterohaemorrhagiae	1∶160
2003	35	Co-agglutination (Icterohaemorrhagiae; Javanica)	≤1∶1280	Icterohaemorrhagiae	1∶160
2003	56	Co-agglutination (Icterohaemorrhagiae; Autumnalis)	≤1∶1280	Icterohaemorrhagiae	1∶160
2003	58	Icterohaemorrhagiae	1∶1280	Icterohaemorrhagiae	1∶320
2004	95	Icterohaemorrhagiae	1∶5160	Co-agglutination (Icterohaemorrhagiae; Ballum)	≤1∶320
2004	22	Co-agglutination (Icterohaemorrhagiae; Autumnalis)	≤1∶1280	Icterohaemorrhagiae	1∶1280
2004	7	Icterohaemorrhagiae	1∶1280	Icterohaemorrhagiae	1∶320
2004	19	Icterohaemorrhagiae	1∶1280	Icterohaemorrhagiae	1∶1280
2005	5	Icterohaemorrhagiae	1∶2560	Icterohaemorrhagiae	1∶160
2005	25	Icterohaemorrhagiae	1∶1280	Icterohaemorrhagiae	1∶320
2005	100	Icterohaemorrhagiae	1∶5120	Icterohaemorrhagiae	1∶1280
2005	6	ND	NA	Ballum	1∶320
2006	82	Icterohaemorrhagiae	1∶640	Icterohaemorrhagiae	1∶160
2006	83	Icterohaemorrhagiae	1∶2560	Icterohaemorrhagiae	1∶160
2006	12	Ballum	1∶2560	Icterohaemorrhagiae	1∶320
2007	28	Co-agglutination (Icterohaemorrhagiae; Ballum)	≤1∶320	Icterohaemorrhagiae	1∶640
2008	26	Icterohaemorrhagiae	1∶1280	Icterohaemorrhagiae	1∶160
2008	99	Icterohaemorrhagiae	1∶2560	Co-agglutination (Icterohaemorrhagiae; Autumnalis)	≤1∶1280
2008	54	Co-agglutination (Icterohaemorrhagiae; Ballum)	≤1∶2560	Icterohaemorrhagiae	1∶640
2008	65	Co-agglutination (Icterohaemorrhagiae; Pomona; Ballum)	≤1∶1280	Icterohaemorrhagiae	1∶640
2009	8	Ballum	1∶1280	Ballum	1∶640
2009	41	ND	NA	Co-agglutination (Icterohaemorrhagiae; Ballum)	≤1∶2560
2010	73	Icterohaemorrhagiae	1∶640	Icterohaemorrhagiae	1∶640
2010	1	Ballum	1∶1280	Co-agglutination (Icterohaemorrhagiae; Ballum)	≤1∶1280
2010	71	ND	NA	Icterohaemorrhagiae	1∶320
2011	68	Co-agglutination (Icterohaemorrhagiae; Javanica)	≤1∶2560	Co-agglutination (Icterohaemorrhagiae; Australis; Ballum)	≤1∶2560
2011	69	Co-agglutination (Icterohaemorrhagiae; Ballum)	≤1∶640	Ballum	1∶160
2011	70	Co-agglutination (Icterohaemorrhagiae; Tarassovi)	≤1∶640	Icterohaemorrhagiae	1∶640

ND: not determined (borderline reactivity). A unique sample was collected from these individuals; the observed titles were below the cut-off (1∶160), value taken by the reference laboratory (IHMT) to endemic areas.

NA: not applicable.

### Association analysis

In order to elucidate which innate immune genes may be involved with human leptospirosis, we genotyped 25 variants among 12 innate immune genes as well as *HLA* class I (–*A* and –*B*) genes. The association analysis for the allelic frequencies (Table S3) show that 3 (12%) out of 25 variants presented susceptibility association values (p<0.05). The alleles -511G in *IL1β* (OR = 1.6, 95% CI 1.08–2.22, p = 0.02), -292T (OR = 1.6, 95% CI 1.02–2.44, p = 0.04) and +3415C (OR = 1.7, 95% CI 1.05–2.61, p = 0.03), both in *CISH*, seem to confer susceptibility to *Leptospira*. The genotypic frequency was also compared (Table 4). Results showed that genotypes -511GG (OR = 1.6, 95% CI 1.01–2.56, p = 0.04) in *IL1β*, +1196CG (OR = 2.0, 95% CI 1.26–3.27, p = 0.003) in *IL12RB1*, -292TA (OR = 1.8, 95% CI 1.06–2.1, p = 0.03) and +3415CG (OR = 1.8, 95% CI 1.08–3.08, p = 0.02), both in *CISH*, have increased risk to leptospirosis. The results for HLA genotyping are presented in Table S3. Data revealed differences between cases and controls in HLA-A*26 (OR = 5.7, 95% CI 1.16–38.2, p = 0.03). The extended *TNF*, *LTA* and *HLA* class I (*–A* and *–B*) haplotype structure was evaluated using genotypic data; however, there was no association with leptospirosis susceptibility (data not shown).

**Table 4:** Significative genotype frequencies and susceptibility to leptospirosis.

Gene: dbSNP	Genotypic frequencies				Association analysis		
	Cases		Controls		Cases *vs* controls		
	N = 97 (%)		N = 470 (%)		OR	(95% CI)	p-value
*IL1β*: rs16944							
**GG**	**54**	**(55.7)**	**206**	**(43.8)**	**1.6**	**(1.01–2.56)**	**0.044**
AG	34	(36.1)	194	(41.1)	0.8	(0.47–1.24)	0.306
AA	9	(9.3)	70	(15.1)	0.6	(0.262–1.27)	0.196
*IL12RB1*: rs401502							
CC	33	(34.0)	190	(40.4)	0.8	(0.47–1.23)	0.288
**CG**	**62**	**(63.9)**	**219**	**(46.6)**	**2.0**	**(1.26–3.27)**	**0.003**
GG	2	(2.1)	61	(13.0)	0.1	(0.02–0.60)	0.003
*CISH*: rs414171							
TT	2	(2.1)	6	(1.3)	1.6	(0.22–9.08)	0.901
**TA**	**32**	**(33.0)**	**103**	**(21.9)**	**1.8**	**(1.06–2.90)**	**0.028**
AA	63	(65.0)	361	(76.8)	0.6	(0.34–0.92)	0.020
*CISH*: rs622502							
CC	1	(1.0)	4	(0.9)	1.2	(0.05–11.65)	1.000
**CG**	**30**	**(30.9)**	**92**	**(19.6)**	**1.8**	**(1.10–3.08)**	**0.019**
GG	66	(68.0)	374	(79.6)	0.5	(0.33–0.91)	0.019

Bold refers to the significant association.

## Discussion

In this retrospective study covering a period of 19 years, we were able to evaluate the seroreactivity against leptospires in 97 unrelated individuals diagnosed with leptospirosis. We found that 46.4% of the participants have circulating anti-*Leptospira* antibodies. This finding indicates that, due to the disease endemicity in the Azores, there is maintenance of leptospires in the animal reservoirs allowing a constant exposure of humans to the infective agent. In fact, the very humid and consistent cool-oceanic climate that Azores archipelago experiences presents the most suitable conditions for the survival and transmission of leptospires. Most likely, repeated contact with the same circulating leptospires leads the immune system to regularly produce anti-*Leptospira* antibodies, attenuating the symptoms in case of a reinfection. Previous studies, performed in the Azores archipelago, identified the serogroups Icterohaemorrhagiae and Ballum to be the most frequent in human [23] and rodent *Leptospira* isolates [13], [14]. This observation is in concordance with data presented in Table 3 that demonstrates the same serogroups among the participants in 2011. Furthermore, all of these participants were asymptomatic for leptospirosis in 2011, despite the fact that they were positive in serology. Moreover, none of the participating individuals have second admissions to the HDES for leptospirosis; although there are no clinical records, we do not exclude the occurrence of a new episode of the disease, only that it has not been recognized as such. For example, individuals 12, 97 and 103 were reinfected with unrelated *Leptospira* serovars, and revealed an absence of symptoms clinically compatible with leptospirosis. These observations led us to hypothesize that the first infection may have acted as a natural live vaccine conferring cross-protection among unrelated *Leptospira* serovars. As far as we know, this is the first retrospective study that reports an immune cross-protection among *Leptospira* serovars in humans; however, it has already been demonstrated in hamsters [4], [24]. Perhaps, in endemic environments, where leptospires have identical circulating serogroups and the seroprevalence is relatively high, an attenuated vaccine could help to prevent leptospirosis in the population at risk. In Cuba, another endemic island for leptospirosis, a trivalent human vaccine is available [25] and the existence of immune cross-protection among vaccine strains of different serovars was ascertained [24]. Other countries, such as Japan and France, also commercialize a monovalent vaccine against leptospirosis with reports of efficacy>70% with little or no side effects [26] and no reports of new reformulation of these vaccines. In the São Miguel Island, the SPIROLEPT vaccine [27], where inactivated *Leptospira* Icterohaemorrhagiae is the principal component, would be the appropriate vaccine for the population at risk, since this was the most frequent serogroup among the 45 participants with positive serology.

In the present study, we were also able to identify three genes that seem to be involved in the susceptibility to leptospirosis infection: *IL1β*, *IL12RB1* and *CISH*. Our data shows that *IL1β* -511GG genotype has increased susceptibility values in cases group. This genotype has also been considered to be a 1.98 higher risk for *H. pylori* eradication failure when compared to -511GA and -511AA genotypes [28]. Moreover, susceptibility to bacteremia within the first year after kidney transplantation was also reported for -511GG genotype [29]. Taken together, these observations support the indication that *IL1β* polymorphisms are involved in susceptibility to bacterial infections, such as leptospirosis. For *IL12RB1*, our data demonstrates that in heterozygosity, the + 1196CG genotype has an increased risk in leptospirosis cases (p = 0.003), which contrast with the + 1196GG genotype that confers a protective effect (p = 0.003). Other polymorphisms in *IL12RB1* have been associated with *M. tuberculosis* [30], [31], inflammatory bowel disease [32] and psoriasis [33]. However, as far as we know, this is the first association of rs401502 variant with an infectious disease. Nevertheless, the mechanism by which *IL1β* and *IL12RB1* mediates protection against human leptospirosis is unknown. It would be of interest to assess the functional effect of these polymorphisms in the phenotype behaviour of leptospirosis patients.

We evaluated the effect of *CISH* variants on leptospirosis susceptibility and the data revealed that, in heterozygosity, -292TA and + 3415CG are risk genotypes in leptospirosis cases. Although there is only one subject for the homozygous risk genotype for both variants, lacking the power to statistically demonstrate this difference, our results indicate that a carrier of these polymorphisms has an increased susceptibility to *Leptospira* infection. CISH (Cytokine-inducible SH2-containing protein) controls the signalling of a variety of cytokines including erythropoietin (EPO), interleukin-2 (IL-2), IL-3 and granulocyte-macrophage colony-stimulating factor (GM-CSF) [34], [35]. In 2010, Khor and colleagues [11] found a relation between *CISH* variants and susceptibility to bacteremia, malaria and tuberculosis, with rs414171 accounting for most of the association signal. In mice, strong expression of *CISH* was observed in the kidney, lung and liver [36], the major and primary affected organs during a leptospirosis infection. This suggests that *CISH* plays a clinically-relevant role that might provide new strategies for controlling infectious agents and inflammatory diseases. Therefore, it is strongly recommended to further investigate the role of *CISH* expression in leptospirosis patients. Another analysis, performed in Khor's study [11], found that the position −292 of the *CISH* promoter was the most highly associated, increasing the overall risk of infectious disease by at least 18% among persons carrying this variant allele. We conducted the same analysis with our data, but no increased risk was observed for leptospirosis cases on São Miguel Island (data not shown).

In an effort to investigate the effect of *TLR* variants, especially *TLR2* and *TLR4*, we genotyped the most studied variants in a high risk population exposed to leptospirosis. Results obtained for the *TLR* variants revealed no association to leptospirosis. In what concerns leptospirosis, the Toll-like receptors are probably the most well-known receptors. These receptors have been studied in animal models – mice and hamster – as a route to find human susceptibility loci to leptospirosis. A recent study, performed by Xue and colleagues [37], used comparative transcriptomics to explain different immune responses to *Leptospira* between murine peritoneal macrophages (MPMs) and human peripheral blood monocytes (HBMs). It was evidenced that *TLR2* and *TLR4* gene expression had no significant regulation in MPMs nor in HBMs, which validates the results obtained here.

Our data analysis of *HLA* class I genes (*–A* and *–B*) suggest an association between HLA-A*26 and an increased risk to leptospirosis. This result does not validate previously reported associations of HLA-A*24, HLA-A*31 and HLA-B*08 alleles with leptospirosis performed in the Azorean island of Terceira [6]. However, due to (1) the limited number of individuals carrying these particular combinations of specific HLA molecules and (2) large confidence interval values obtained from statistical analysis, this may not be a reliable association risk. Extended *HLA–A* and *–B* haplotypes did not reveal any association either. Interestingly, in Xue's study [37], the genes involved in antigen processing and presentation pathways in MPMs and HBMs were mainly down-regulated, and the down regulations in HBMs were more significant than those in MPMs. Taken together, these findings suggest that probably HLA genes are not directly associated with host defence against *Leptospira*.

In the present study, some limitations emerged due to the sample size and study design. Since this is a retrospective study, it was difficult to get all the participants to come back to the hospital, especially those who had the illness more than 10 years ago, mainly for three reasons: a) live far from the hospital, b) denied that they had the illness, or c) some of them already died. Another limitation in this study was the control group. Since they were anonymous individuals, we were not able to get serum samples and evaluate the seroreactivity. For this reason, it would be of interest to assess the seroreactivity against *Leptospira* in the general population of São Miguel Island to determine the global incidence of asymptomatic leptospirosis. Although the sampling size was a limitation, we were able to demonstrate statistically significant differences in the distribution of genotypes in terms of infection, between cases and controls, thus suggesting an association with the illness. Moreover, the variants here found are reported in studies that used larger samples.

In conclusion, the study here presented suggests some degree of long-term protection against leptospires with an attenuation of symptoms in case of reinfection. Furthermore, the evidence of cross-protection among *Leptospira* serovars could be representative of the human immune response to a viable vaccine against *Leptospira*. Finally, genetic findings revealed *IL1β*, *IL12RB1* and *CISH* to be involved in susceptibility to leptospirosis infection. The functional effects of these genes should be further investigated, as well as the gene expression of human leptospirosis patients.

## Supporting Information

Table S1 **Genetic information of the 14 selected candidate genes of the innate immune system.**
(DOC)

Table S2 **Primers and probes used for variants genotyping by singleplex PCR methods.**
(DOC)

Table S3 **Allele frequencies and risk variants associated with susceptibility to leptospirosis.**
(DOC)
