# Supplementary material for: Human Leptospirosis: Seroreactivity and Genetic Susceptibility in the Population of São Miguel Island (Azores, Portugal)
Source: PLoS One. 2014 Sep 25;9(9):e108534. doi: 10.1371/journal.pone.0108534 (PMC4177921; doi:10.1371/journal.pone.0108534)
Supplement: Table S1 — Genetic information of the 14 selected candidate genes of the innate immune system. (DOC) [file pone.0108534.s001.doc]

| **Supplementary Table S1**. Genetic information of the 14 selected candidate genes of the innate immune system. | | | | | |
| --- | --- | --- | --- | --- | --- |
| **Gene** |  |  | **Variants** |  |  |
| **Name** | **Symbol** | **Chr band** | **dbSNP** | **Position** | **Ancestral allele > Variant allele^a^** |
| *Interleukin 1 alpha* | *IL1α* | 2q14 | rs1800587 | -889 | C>T |
| *Interleukin 1 beta* | *IL1β* | 2q14 | rs16944 | -511 | A>G |
| *Interleukin 6* | *IL6* | 7p21 | rs1800797 | -596 | G>A |
|  |  |  | rs1800795 | -174 | G>C |
| *Interleukin 10* | *IL10* | 1q31-q32 | rs1800896 | -1082 | A>G |
|  |  |  | rs1800871 | -819 | C>T |
|  |  |  | rs1800872 | -592 | C>A |
| *Interleukin 12 receptor, beta 1* | *IL12RB1* | 19p13.1 | rs401502 | +1196 | C>G |
| *Toll-like receptor 2* | *TLR2* | 4q32 | rs4696480 | -16933 | T>A |
|  |  |  | rs121917864 | +2029 | C>T |
|  |  |  | rs5743708 | +2259 | G>A |
|  |  |  | rs201786064 | -196 to -174^b^ | ins>del |
| *Toll-like receptor 4* | *TLR4* | 9q33.1 | rs4986790 | +896 | A>G |
|  |  |  | rs4986791 | +1196 | C>T |
| *Cytokine-inducible SH2-containing protein* | *CISH* | 3p21.3 | rs414171 | -292 | T>A |
|  |  |  | rs6768330 | -163 | A>C |
|  |  |  | rs2239751 | +1320 | A>C |
|  |  |  | rs622502 | +3415 | C>G |
| *Toll-like receptor 9* | *TLR9* | 3p21.3 | rs187084 | -1486 | C>T |
|  |  |  | rs5743836 | -1237 | C>T |
| *Cluster of differentiation 14* | *CD14* | 5q22-q32 | rs2569190 | -260 | G>A |
|  |  |  | rs2569191 | -159 | T>C |
| *Lymphotoxin alpha (TNF superfamily, member 1)* | *LTA* | 6p21.3 | rs2844482^c^ | -294 | G>A |
| *Tumor necrosis factor* | *TNF* | 6p21.3 | rs1800629 | -308 | G>A |
|  |  |  | rs361525 | -238 | G>A |
| *Human leukocyte antigen –* A | *HLA–A* | 6p21.3 |  |  |  |
| *Human leukocyte antigen –* B | *HLA–B* | 6p21.3 |  |  |  |
| ^a^According to dbSNP Reference SNP(refSNP) | | | | | |
| ^b^This indel variant is located in the 5’UTR. | | | | | |
| ^c^This SNP is in complete LD (r^2^ = 1.0) with rs1800630, located on *TNF* gene. | | | | | |
