# Supplementary material for: Human Leptospirosis: Seroreactivity and Genetic Susceptibility in the Population of São Miguel Island (Azores, Portugal)
Source: PLoS One. 2014 Sep 25;9(9):e108534. doi: 10.1371/journal.pone.0108534 (PMC4177921; doi:10.1371/journal.pone.0108534)
Supplement: Table S2 — Primers and probes used for variants genotyping by singleplex PCR methods. (DOC) [file pone.0108534.s002.doc]

| **Supplementary Table S2.** Primers and probes used for variants genotyping by singleplex PCR methods. | | | | |
| --- | --- | --- | --- | --- |
| **Gene** | **dbSNP** | **Assay type** | **Primer (5’→3’)** | **Probe (5’→3’)** |
| *IL10* | rs1800871 | Taqman® | C___1747362_10 | – |
| *TLR2* | -196 to -174 del | In house | Fw – 5′-6FAM-CTCGGAGGCAGCGAGAAA-3′  Rv - 5′-CTGGGCCGTGCAAAGAAG-3′ |  |
| *CISH* | rs414171 | In house | Fw - CCCTCTGGGTAGCTTCAGC  Rv - CCTGAGCAGTGAAAGGAAATACT | FAM - ACCGCTCGGCTCCACCT  VIC - ACCGCACGGCTCCACC |
|  | rs6768330 | In house | Fw - CTAAGTAGAGACAATGTCCAGCAGGT  Rv - AAGGTTGAAGACTAACCCAAGATCTG | FAM - CCTGCCGCTTCACAACG  VIC - TGCCTGCCTCTTCACAACG |
|  | rs2239751 | Taqman® | C___3142857_10 | – |
|  | rs622502 | Taqman® | C___34142855_10 | – |
| *TLR9* | rs5743836 | Taqman® | C__32645383_10 | – |
| *LTA* | rs2844482 | In house | Fw - GCCAAGGGTGCAGAGATGTT  Rv - TCAGGGGAATCGTGGGCT | FAM - TCAACCACCTCCTCTCTGAATTG  VIC - TCAACTACCTCCTCTCTGAATTGACTG |
| *TNF* | rs1800629 | Taqman® | C___7514879_10 | – |
|  | rs361525 | Taqman® | C___2215707_10 | – |
| *LTA, lymphotoxin-alpha* (*TNF superfamily, member 1*); *CISH, cytokine inducible SH2-containing protein*; *TNF*, *tumor necrosis factor*; *TLR2, toll-like receptor 2,TLR9, toll-like receptor 9; IL10, interleukin 10.* | | | | |
