# Supplementary material for: Human Leptospirosis: Seroreactivity and Genetic Susceptibility in the Population of São Miguel Island (Azores, Portugal)
Source: PLoS One. 2014 Sep 25;9(9):e108534. doi: 10.1371/journal.pone.0108534 (PMC4177921; doi:10.1371/journal.pone.0108534)
Supplement: Table S3 — Allele frequencies and risk variants associated with susceptibility to leptospirosis. (DOC) [file pone.0108534.s003.doc]

| **Supplementary Table S3.**  Allele frequencies and risk variants associated with susceptibility to leptospirosis. | | | | | | | | |
| --- | --- | --- | --- | --- | --- | --- | --- | --- |
| **Gene** | **dbSNP (allele)** | **Allele frequency** | | | | **Association analysis** | | |
|  |  | **Patients** | | **Controls** | | **Patients *vs* controls** | | |
|  |  | **2N=194 (%)** | | **2N=940 (%)** | | **OR** | **(95% CI)** | **p-value** |
| *IL1α* | rs1800587 (C) | 120 | (61.9) | 625 | (66.5) | 0.8 | (0.59-1.14) | 0.25 |
| ***IL1β*** | **rs16944 (G)** | **143** | **(73.7)** | **606** | **(64.5)** | **1.6** | **(1.08-2.22)** | **0.02** |
| *IL6* | rs1800797 (G) | 132 | (68.0) | 604 | (64.3) | 1.2 | (0.84-1.67) | 0.36 |
|  | rs1800795 (G) | 134 | (69.1) | 623 | (66.3) | 1.1 | (0.80-1.61) | 0.50 |
| *IL10* | rs1800896 (A) | 121 | (62.4) | 551 | (58.6) | 1.2 | (0.84-1.63) | 0.37 |
|  | rs1800871 (C) | 137 | (70.6) | 675 | (71.8) | 0.9 | (0.66-1.35) | 0.81 |
|  | *rs1800872* (C) | 137 | (70.6) | 678 | (72.1) | 0.9 | (0.65-1.33) | 0.74 |
| *IL12RB1* | rs401502 (C) | 128 | (66.0) | 599 | (63.7) | 1.1 | (0.79-1.55) | 0.61 |
| *TLR2* | rs4696480 (T) | 86 | (44.3) | 397 | (42.2) | 1.1 | (0.79-1.51) | 0.65 |
|  | rs121917864 (C) | 194 | (100.0) | 939 | (99.9) | Inf | (0.01-Inf) | 1.00 |
|  | rs5743708 (G) | 193 | (99.5) | 932 | (99.2) | 1.7 | (0.21-35.5) | 0.97 |
|  | -196 to -174 (ins) | 156 | (80.4) | 769 | (81.8) | 0.9 | (0.61-1.38) | 0.72 |
| *TLR4* | rs4986790 (A) | 184 | (94.9) | 880 | (93. 6) | 1.3 | (0.61-2.66) | 0.63 |
|  | rs4986791 (C) | 185 | (95.4) | 878 | (93.4) | 1.5 | (0.68-3.19) | 0.39 |
| ***CISH*** | **rs414171 (T)** | **35** | **(18.0)** | **115** | **(12.2)** | **1.6** | **(1.02-2.44)** | **0.04** |
|  | rs6768330 (A) | 163 | (84.0) | 818 | (87.0) | 0.8 | (0.50-1.23) | 0.32 |
|  | rs2239751 (A) | 187 | (96.4) | 925 | (98.4) | 0.4 | (0.16-1.19) | 0.12 |
|  | **rs622502 (C)** | **32** | **(16.5)** | **100** | **(10.6)** | **1.7** | **(1.05-2.61)** | **0.03** |
| *TLR9* | rs187084 (C) | 89 | (45.9) | 369 | (39.3) | 1.3 | (0.95-1.81) | 0.10 |
|  | rs5743836 (C) | 24 | (12.4) | 145 | (15.4) | 0.8 | (0.47-1.26) | 0.33 |
| *CD14* | rs2569190 (G) | 111 | (57.2) | 525 | (55.9) | 1.1 | (0.77-1.46) | 0.79 |
|  | rs2569191 (T) | 107 | (55.2) | 539 | (57.3) | 0.9 | (0.66-1.26) | 0.63 |
| *LTA* | rs2844482 (G) | 140 | (71.4) | 693 | (73.7) | 0.9 | (0.65-1.33) | 0.72 |
| *TNF* | rs1800629 (G) | 169 | (87.1) | 796 | (84.7) | 1.2 | (0.76-1.98) | 0.45 |
|  | rs361525 (G) | 183 | (93.8) | 875 | (93.1) | 1.2 | (0.62-2.53) | 0.64 |
| HLA-A | | | | | | | | |
| A*01 |  | 28 | (14.4) | 32 | (15.1) | 1.0 | (0.53-1.70) | 0.96 |
| A*02 |  | 42 | (21.7) | 53 | (25.0) | 0.8 | (0.51-1.35) | 0.49 |
| A*03 |  | 16 | (8.3) | 20 | (9.4) | 0.9 | (0.41-1.81) | 0.81 |
| A*11 |  | 13 | (6.7) | 9 | (4.2) | 1.6 | (0.63-4.22) | 0.38 |
| A*23 |  | 4 | (2.1) | 4 | (1.9) | 1.1 | (0.23-5.28) | 1.00 |
| A*24 |  | 21 | (10.8) | 29 | (13.7) | 0.8 | (0.40-1.45) | 0.47 |
| A*25 |  | 1 | (0.5) | 1 | (0.5) | 1.1 | (0.03-40.2) | 1.00 |
| **A*26** |  | **10** | **(5.2)** | **2** | **(0.9)** | **5.7** | **(1.16-38.22)** | **0.03** |
| A*29 |  | 11 | (5.7) | 14 | (6.6) | 0.9 | (0.35-2.05) | 0.85 |
| A*30 |  | 5 | (2.6) | 7 | (3.3) | 0.8 | (0.21-2.77) | 0.89 |
| A*31 |  | 7 | (3.6) | 5 | (2.4) | 1.6 | (0.44-5.73) | 0.65 |
| A*32 |  | 9 | (4.6) | 13 | (6.1) | 0.8 | (0.29-1.91) | 0.66 |
| A*33 |  | 6 | (3.1) | 6 | (2.8) | 1.1 | (0.31-3.91) | 1.00 |
| A*34 |  | 1 | (0.5) | 0 | (0.0) | Inf | (0.06-Inf) | 0.97 |
| A*66 |  | 2 | (1.0) | 1 | (0.5) | 2.2 | (0.16-61.7) | 0.94 |
| A*68 |  | 15 | (7.7) | 15 | (7.1) | 1.1 | (0.49-2.46) | 0.95 |
| A*74 |  | 1 | (0.5) | 0 | (0.0) | Inf | (0.06- Inf) | 0.97 |
| A*80 |  | 2 | (1.0) | 1 | (0.5) | 2.2 | (0.16-61.7) | 0.94 |
| HLA-B | | | | | | | | |
| B*07 |  | 15 | (15.5) | 14 | (6.6) | 1.2 | (0.52-2.68) | 0.80 |
| B*08 |  | 17 | (17.5) | 29 | (13.7) | 0.6 | (0.31-1.19) | 0.16 |
| B*13 |  | 3 | (3.1) | 1 | (0.5) | 3.3 | (0.31-83.4) | 0.55 |
| B*14 |  | 13 | (13.4) | 15 | (7.1) | 0.9 | (0.41-2.16) | 1.00 |
| B*15 |  | 14 | (14.4) | 11 | (5.2) | 1.4 | (0.59-3.45) | 0.52 |
| B*18 |  | 14 | (14.4) | 11 | (5.2) | 1.4 | (0.59-3.45) | 0.52 |
| B*27 |  | 4 | (4.1) | 9 | (4.2) | 0.5 | (0.12-1.72) | 0.33 |
| B*35 |  | 17 | (17.5) | 13 | (6.1) | 1.5 | (0.66-3.32) | 0.41 |
| B*37 |  | 2 | (2.1) | 3 | (1.4) | 0.7 | (0.08-5.39) | 1.00 |
| B*38 |  | 4 | (4.1) | 3 | (1.4) | 1.5 | (0.27-8.36) | 0.91 |
| B*39 |  | 3 | (3.1) | 2 | (0.9) | 1.7 | (0.22-14.2) | 0.92 |
| B*40 |  | 7 | (7.2) | 6 | (2.8) | 1.3 | (0.38-4.40) | 0.87 |
| B*41 |  | 1 | (1.0) | 5 | (2.4) | 0.2 | (0.009-1.90) | 0.26 |
| B*44 |  | 28 | (28.9) | 33 | (15.6) | 0.9 | (0.51-1.63) | 0.86 |
| B*45 |  | 7 | (7.2) | 2 | (0.9) | 3.9 | (0.74-27.7) | 0.14 |
| B*48 |  | 2 | (2.1) | 0 | (0.0) | Inf | (0.27- Inf) | 0.44 |
| B*49 |  | 7 | (7.2) | 11 | (5.2) | 0.7 | (0.23-1.95) | 0.59 |
| B*50 |  | 2 | (2.1) | 7 | (3.3) | 0.3 | (0.04-1.62) | 0.22 |
| B*51 |  | 15 | (15.5) | 14 | (6.6) | 1.2 | (0.52-2.68) | 0.80 |
| B*52 |  | 1 | (1.0) | 0 | (0.0) | Inf | (0.063- Inf) | 0.97 |
| B*53 |  | 3 | (3.1) | 5 | (2.4) | 0.7 | (0.12-3.17) | 0.81 |
| B*55 |  | 2 | (2.1) | 4 | (1.9) | 0.5 | (0.07-3.47) | 0.76 |
| B*56 |  | 1 | (1.0) | 0 | (0.0) | Inf | (0.06-Inf) | 0.97 |
| B*57 |  | 7 | (7.2) | 9 | (4.3) | 0.8 | (0.28-2.53) | 0.94 |
| B*58 |  | 5 | (5.2) | 3 | (1.4) | 1.8 | (0.38-9.86) | 0.63 |
| Bold refers to the significant association. | | | | | | | | |
